# The Associations Between Chronic Active Lesions and White Matter Disease: A 7 Tesla Imaging Study

**DOI:** 10.1002/acn3.70101

**Published:** 2025-09-16

**Authors:** Ellie McCluey, Ahmad A. Toubasi, Jiacheng Wang, Habeeb F. Kazimuddin, Taegan Vinarsky, Caroline Gheen, Carynn Koch, Zachery Rohm, Bryan Hernandez, Margareta A. Clarke, Rachael Cheek, Rozita Khalili, Chaoyang Jin, Victoria Lim, John Kramer, Junzhong Xu, Ipek Oguz, Francesca Bagnato

**Affiliations:** ^1^ Neuroimaging Unit, Neuroimmunology Division, Department of Neurology Vanderbilt University Medical Center (VUMC) Nashville Tennessee USA; ^2^ Vanderbilt University (VU) College of Arts and Science Nashville Tennessee USA; ^3^ VU Department of Computer Science Nashville Tennessee USA; ^4^ National Institute of Neurological Disorders and Stroke (NINDS) National Institutes of Health (NIH) Bethesda Maryland USA; ^5^ Medical Scientist Program VU School of Medicine Nashville Tennessee USA; ^6^ College of Science, Department of Biological Sciences University of Texas at El Paso El Paso Texas USA; ^7^ VU School of Medicine, Department of Biochemistry Nashville Tennessee USA; ^8^ Meharry Medical College, School of Medicine Nashville Tennessee USA; ^9^ Vanderbilt Institute of Imaging Science, Department of Radiology and Radiological Science, VUMC Nashville Tennessee USA; ^10^ Neuroimmunology Division, Department of Neurology, VUMC Nashville Tennessee USA; ^11^ Department of Neurology, Nashville VA Medical Center TN Valley Healthcare System Nashville Tennessee USA

**Keywords:** chronic inflammation, demyelination, normal appearing white matter, paramagnetic rim lesions

## Abstract

**Background:**

The relationship between paramagnetic rim lesions (PRLs) and surrounding normally appearing white matter (NAWM) disease, potentially contributory to the associations seen between PRLs and clinical impairment, is underexplored.

**Objectives:**

To assess whether PRLs correlate with a greater degree of NAWM injury in early MS.

**Methods:**

PRLs were identified on susceptibility weighted imaging (SWI) of 68 newly diagnosed patients. Each PRL was paired with an anatomically matched contralateral non‐PRL (nPRL) from the same participant. Quantitative magnetization transfer imaging derived macromolecular‐to‐free pool‐size ratio (PSR) and relaxation rate of the free water pool (R_1f_) values were extracted and compared between PRLs and nPRLs, NAWM surrounding PRLs and nPRLs, and whole brain WM lesions and NAWM of PRL+ and PRL− people with MS (pwMS).

**Results:**

PSR and R_1f_ (*p* ≤ 0.028) values were lower in PRLs compared to nPRLs, but there were no differences in PSR and R_1f_ (*p* ≥ 0.824) values between peri‐PRLs and peri‐nPRLs NAWM ROIs. PRL+ pwMS had similar PSR and R_1f_ (*p* ≥ 0.267) of the whole brain NAWM, similar WM lesions PSR (*p* = 0.764) but lower R_1f_ (*p* = 0.030) values.

**Conclusions:**

In the early stages of MS, there is no association between PRLs and surrounding NAWM degree of injury.

## 1 Introduction

Paramagnetic rim lesions (PRLs) are a biomarker of chronic inflammation and microglia activation in people with multiple sclerosis (MS) [1]. Irrespective of the disease phase, PRLs are associated with poorer disease outcomes, both clinically and radiologically [1, 2].

Extensive literature based on histology and quantitative magnetic resonance imaging (MRI) data supports the notion that MS pathology extends beyond focal lesions to areas of the so‐called normal appearing white matter (NAWM). Specifically, reduced axonal density [3, 4], activated microglia [5, 6], increased oxidative damage [7, 8], and altered mitochondrial morphology [9] have all been detected in NAWM of people with MS (pwMS). Studies have also shown that NAWM areas surrounding lesions tend to be more injured than those distant from lesions [10] due to the anterograde and retrograde Wallerian degeneration [11] exerted by the truncated axons within the lesions' cores. Injury of the NAWM is relevant because it is associated with cognitive and physical impairments of pwMS [12].

Selective inversion recovery quantitative magnetization transfer (SIR‐qMT) is an in‐house developed qMT method [13, 14, 15] that delivers more accurate and clinically correlated estimates of myelin content [15, 16, 17, 18] as we demonstrated with animal‐based [15], histology [15, 17] and clinical [16, 17, 18] work in our group. SIR‐qMT produces the macromolecular‐to‐free water pool‐size ratio (PSR) and the spin–lattice relaxation rate of free water (R_1f_). PSR estimates the degree of myelin integrity by measuring the ratio of the bound macromolecular protons to the free water ones. R_1f_ provides insights into the microenvironment, indirectly assessing water accumulation secondary to tissue loss, and is additionally sensitive to iron content [13, 14].

In this 7 Tesla (7 T)‐MRI based study, we focus on a cohort of treatment‐naïve people with newly diagnosed MS (pwMS hereafter), clinically isolated syndrome (pwCIS) or radiologically isolated syndrome (pwRIS). We asked whether the presence of PRLs relates to a greater degree of NAWM tissue injury. We addressed this question using a lesion‐ and a subject‐level approach. With lesion level analyses, we compared PSR and R_1f_ values between the cores of PRLs to that of non‐PRLs (nPRLs) and subsequently compared and correlated these values with those measured in the respective surrounding NAWM. With subject level analyses, we examined whether whole brain WM lesions and NAWM of patients with PRLs exhibit lower PSR and R_1f_ values compared to those of patients without PRLs.

## 2 Materials and Methods

### 2.1 Study Design and Cohort

This prospective, cross‐sectional study was conducted at the Vanderbilt University Medical Center (VUMC) MS clinic upon the Institutional Review Board approval and signed consent from each participant. Sixty‐eight newly diagnosed treatment‐naïve pwCIS [19], pwRIS [20] and pwMS [19] were consecutively enrolled and underwent a 7 T MRI and a neurological evaluation using the Expanded Disability Status Scale (EDSS) [21], the 9‐hole peg test (9‐HPT) [22, 23], and the timed 25‐ft walking test (T25‐FW) [22].

The following were considered exclusion criteria: contraindication for an MRI, history or presence of other central nervous system autoimmune, neoplastic, infectious, or vascular conditions, diagnosis of uncontrolled hypertension, diabetes, hyperlipidemia, and current or past use of illicit drugs. Except for five individuals, all participants had not used steroids for at least 30 days. Furthemore, excpet for two participants, none has been previoulsy exposed to disease modifying agents.

### 2.2 MRI Acquisition Protocol

A 7 T whole body MRI Philips Achieva scanner equipped with a two‐channel volume transmit and a 32‐channel receive‐only head coil (Nova Medical, Wilmington, MA) was used for scanning. The MRI protocol included a 3‐dimensional (3D) magnetization prepared 2 rapid gradient echoes (MP_2_RAGE), a 3D T_2_‐weighted (T_2_‐w) fluid attenuated inversion recovery image (FLAIR), a 3D single echo gradient echo (SE‐GRE) from which susceptibility weighted imaging (SWI) was derived, and a 2D SIR‐qMT from which PSR and R_1f_ were computed. Except for the MP_2_RAGE, which was acquired in the sagittal plane, all other scans were acquired axially. Details of pulse sequence parameters have been previously reported [24, 25]. To assess for contrast enhancing lesions (CELs), the first 27 subjects received a post‐gadolinium diethylenetriamine penta‐acetic acid (Gd‐DPTA) MP_2_RAGE scan at 7 T. In 22 subjects, CELs were identified on a 3 T MPRAGE scan obtained in the setting of another ongoing research study within a median of 5.5 days [0–8.5] from the 7 T scan. The 1 mm isotropic 3 T MPRAGE sequence had an echo time of 4.6 milliseconds and an inversion recovery time of 9.0 milliseconds. In the remaining 19 subjects, CELs were assessed using a clinical scan performed by the treating physician to assess disease activity for clinical purposes. The clinical scan included an axial T_2_‐w turbo spin echo or fast field echo and T_2_‐w FLAIR along with a T_1_‐w post‐Gd‐DPTA sequence used to assess CELs. The magnet field varied depending on the setting where the MRI was performed. Of the subjects imaged at the VUMC hospital, five underwent a 1.5 T MRI, and seven were scanned using a 3 T MRI. The remaining people (*n* = 7) were scanned in institutions outside ours. The median number of days between the clinical T_1_‐w post‐Gd‐DPTA and our 7 T research scan was 35 [16–48]. Irrespective of the acquisition method, post‐Gd‐DPTA was administered intravenously (IV) at a dose of 0.1 mmol/kg of body weight.

### 2.3 MRI Post‐Processing

MP_2_RAGE and SWI were computed as elsewhere reported [24].

#### 2.3.1 Image Registration

The T_2_‐w FLAIR images were registered on the SWI using a linear registration tool with mutual information as the cost function in the FLIRT/FSL suite [26]. MP_2_RAGE images were then registered to these co‐registered FLAIR images through ANTs [27], using the Mattes metric as the cost function.

#### 2.3.2 Whole Brain NAWM Segmentation

FreeSurfer [28] (version 7.0) was used to perform whole‐brain segmentation, applying the Desikan‐Killiany [29] and Destrieux [30] atlases. Preprocessing began with motion correction and skull stripping, followed by intensity normalization. To correct for field inhomogeneities and refine pial surface detection, N4BiasFieldCorrection [31] was applied to both MP_2_RAGE and T_2_‐w FLAIR images prior to segmentation. The standard recon‐all pipeline was used to segment WM and gray matter structures and generate cortical and subcortical parcellations based on the Desikan‐Killiany atlas [29]. The hires flag was enabled for images with a native resolution below 1 mm^3^ to allow for submillimeter surface modeling. NAWM was defined as WM regions excluding any lesion masks and was extracted from the segmented output. Each segmentation was visually inspected by two investigators (E.M.C. and H.F.K.) to confirm anatomical accuracy and ensure proper delineation of NAWM. These refined NAWM masks were then used for subsequent volumetric and quantitative analyses.

#### 2.3.3 Brain Volume Estimation

Brain volume normalization was performed using the Structural Image Evaluation with Normalization of Atrophy Cross‐sectional (SIENAX) [32] in FSL. To ensure precise measurements of NAWM, manually traced WM lesion masks were subtracted from the SIENAX segmentation results. Given the potential for segmentation errors in the posterior fossa, analyses were restricted to brain regions located above the mammillary bodies, determined by setting an axial‐plane boundary.

### 2.4 Image Analyses

#### 2.4.1 WM Lesions Identification, Delineation and Volume Computation

Before WM lesions masking, signal inhomogeneities of the T_2_‐w FLAIR images were corrected using the N4BiasFieldCorrection. Visualization, graphical, and statistical tools in Medical Image Processing, Analysis and Visualization (MIPAV) [33] software were used to perform image analysis. T_2_‐lesions were identified as WM hyperintensities on T_2_‐w FLAIR images [3, 24, 25, 26, 27, 28, 29, 30, 31, 32, 33, 34] and manually delineated by several investigators (H.F.K., B.H., M.A.C., A.A.T., E.M.). The senior author (F.B.) FB reviewed all the T_2_‐lesions masks and identified all the CELs, which were subsequently excluded from all the analyses.

#### 2.4.2 PRLs Assessment

To identify PRLs, co‐registered T_2_‐w FLAIR images and SWI maps were used in conjunction with post‐Gd‐DTPA sequences. We used a default second order B_0_ shimming provided by Philips, which satisfactorily eliminates the undesired inhomogeneities of the SE‐GRE. Some, expecialy the ones observed in the posterior fossa may remain uncorrectable, and for this reason, lesions located in those areas were not included in the analysis. Two investigators (H.F.K., A.A.T.) identified PRLs, followed by group discussions (H.F.K., B.H., K.C., Z.R.), with final approval from a senior investigator (F.B.) to resolve discrepancies. In adherence to the North America Imaging in MS consensus [1], PRLs were identified as non‐CELs characterized by a hyperintense core on T_2_‐w FLAIR and a surrounding paramagnetic rim on SWI. The paramagnetic rim had to appear on two or more consecutive slices, covering at least two‐thirds of the core on the clearest slice. Fused PRLs, for example, two PRLs coalescing into one T_2_‐lesion, were treated as a single lesion, as we previously detailed [24]. Individuals with one or more PRLs visible on SWI were classified as PRL+.

#### 2.4.3 Perilesional NAWM Assessment

Regions of interest (ROIs) in the NAWM adjacent to PRLs and their corresponding nPRLs were delineated using graphic tools available in MIPAV.

**FIGURE 1:**
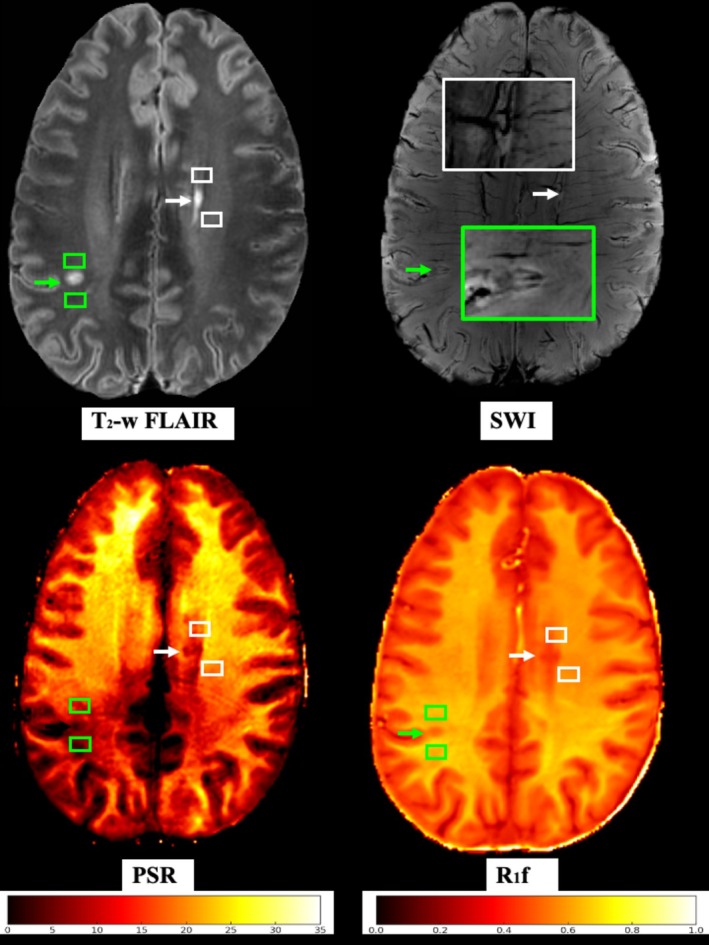
Paramagnetic rim lesions and normal appearing white matter regions of interest. The green arrow points toward a paramagnetic rim lesion (PRL) on the T_2_‐weighted fluid attenuated inversion recovery image (T_2_‐w FLAIR) and susceptibility weighted images (SWI), while the white arrow points toward a non‐PRL (nPRL). Green and white rectangles anteriorly and posteriorly to the two lesions indicate the corresponding normal appearing white matter (NAWM) regions of interest (ROIs). The same lesions and NAWM ROIs are shown on the heat maps of the macromolecular‐to‐free water pool‐size ratio (PSR) and the spin–lattice relaxation rate of free water (R_1f_).

For each PRL, an anatomically matched contralateral nPRL was identified within three slices above or below the corresponding PRL. Care was taken to ensure that PRLs and matched nPRL were approximately the same size, equidistant from surrounding lesions, and located in the same WM regions. Thereafter, two ROIs were manually drawn, immediately anterior and posterior to each PRL and nPRL core. For PRLs visible across multiple consecutive slices, ROIs were placed on the slice exhibiting the maximal lesion visibility. We placed each NAWM ROI as close as possible to the related PRL or nPRL, but care was taken that these ROIs were not touching any lesions and were as distant as possible from the gray matter. Given the heterogeneity of MS, these features varied from patient to patient. Furthermore, a minimum volume threshold of 21 mm^3^, corresponding to 10 voxels of the acquired SIR‐qMT, was maintained for all ROIs to avoid computational biases from the partial volume effect. Figure 1 shows examples of PRL, nPRL, and NAWM ROI identification and delineation process.

A single investigator (E.M.) identified and delineated all the NAWM ROIs, which were subsequently reviewed by a second investigator (A.A.T.) for accuracy and discussed with the last author (F.B.) as appropriate. To ensure the reliability of the results irrespective of the rater and associated inter‐rater variability, we evaluated the inter‐rater correlation between two investigators (E.M. and V.K.) on 24 T_2_‐lesions (12 PRLs and 12 nPRLs) randomly picked.

#### 2.4.4 PSR and R_1f_ Data Extraction

T_2_‐lesion, whole brain and ROI based NAWM masks were overlaid on the co‐registered PSR and R_1f_ maps, and corresponding values were derived. For these analyses, PRLs were removed from the T_2_‐lesion masks.

### 2.5 Statistical Analyses

Counts with percentage and mean ± standard deviation (SD) were used to report categorical and continuous variables, respectively.

A paired sample *t*‐test and the inter‐class correlation (ICC) analysis were employed to assess inter‐rater agreement in PSR and R_1f_ values of the NAWM ROIs generated by the first rater (E.M.) and the second rater (V.K.). Mean differences (MD) and associated 95% confidence intervals (95% CI) were used to report the results of the paired sample *t*‐test, while the Cronbach alpha (*α*) coefficient was used to report the results of the ICC analysis.

Paired sample *t*‐tests were also used to assess differences in PSR and R_1f_ values between PRLs and matching nPRLs, and between either lesion type and their corresponding NAWM ROI. MD and corresponding 95% CI are reported. Linear regression analysis was used to correlate PSR and R_1f_ of PRLs and nPRLs with their corresponding NAWM. A subsequent analysis of variance (ANOVA) between the models was done to compare the strength of these correlations.

An independent sample *t*‐test was used to compare PSR and R_1f_ values of the whole brain NAWM and WM lesions between patients with and without PRLs.

A *p* value < 0.050 was considered statistically significant for all the analyses. The Statistical Package for Social Sciences (SPSS) Statistics for Windows, Version 24.0 (Armonk, NY: IBM Corp) was used for the analyses.

## 3 Results

### 3.1 Study Cohort

A total of 68 subjects, whose demographic, clinical, and MRI features are detailed in Table 1, were enrolled. Of these, four were subsequently eliminated from the anlayses extending beyond PRL identification. One participant lacked the T_2_‐w FLAIR sequence at 7T. In this participant the clinical T2‐w FLAIR was used. One participant had multiple tumefactive lesions and extensive confluent T_2_‐lesions. Two additional subjects were eliminated from the SIR‐qMT analysis due to lack of SIR‐qMT data. Thus, 64 subjects were included in the final analysis.

**TABLE 1:** Demographic and clinical characteristics of the study cohort (*n* = 68).

	PRL− (*n* = 39)	PRL+ (*n* = 29)	*p*
Age (years)	39 ± 10	38 ± 10	0.540
Sex			
Males	14 (35.9%)	12 (41.4)	0.645
Females	25 (64.1%)	17 (58.6)	
Race			
Blacks	3 (7.7%)	2 (6.9)	0.901
Whites	36 (92.3%)	27 (93.1)	
Body mass index	27.98 ± 5.82	28.30 ± 4.96	0.810
Expanded Disability Status Scale score	1.5 ± 1.7	0.9 ± 1.4	0.166
Timed 25‐foot walk time^a^ (seconds)	5.94 ± 3.89	5.38 ± 2.10	0.493
9‐hole peg test dominant hand^a^, ^b^	21.26 ± 4.46	21.73 ± 3.02	0.721
9‐hole peg test non‐dominant hand^a^, ^b^	20.95 ± 2.58	24.29 ± 4.99	0.008
Days from post‐Gd DTPA MRI^b^	20 [15–43]	35 [15–58]	0.631
Disease phenotype			
RIS	2 (5.1%)	3 (10.3%)	0.698
CIS	5 (12.8%)	3 (10.3%)	
MS	32 (82.1%)	23 (79.3%)	
Months since first symptom^b^	24 [3.5–72]	16 [7–48]	0.569
Days from diagnosis^b^	30 [13–40]	30 [15–68]	0.548
Days from last steroids			
< 30 days	3 (7.7%)	2 (6.9%)	0.901
> 30 days	36 (92.3%)	27 (93.1%)	

*Note:* Data are expressed in mean ± standard deviation unless otherwise stated.

Abbreviations: CIS, clinically isolated syndrome; Gd DTPA, gadolinium diethylenetriamine penta‐acetic acid; MRI, magnetic resonance imaging; MS, multiple sclerosis; PRL, paramagnetic rim lesion; RIS, radiologically isolated syndrome.

^a^ Averaged across two trials.

^b^ Median [1st quartile–3rd quartile].

### 3.2 Inter‐Rater Variability Analyses

**FIGURE 2:**
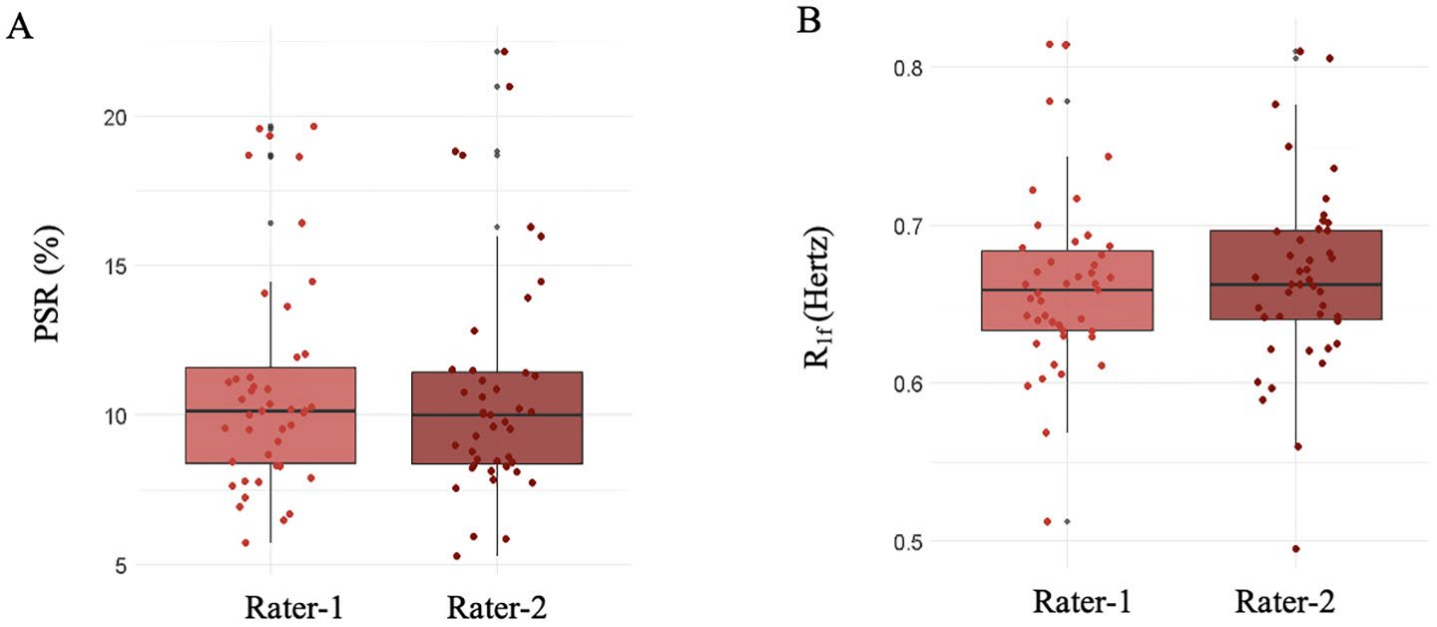
Inter‐rater reliability measurements. The inter‐rater reliability measurements of the macromolecular‐to‐free water pool‐size ratio (PSR, A) and spin–lattice relaxation rate of free water (R_1f_, B) of peri‐paramagnetic rim lesion (PRL) and peri‐non‐PRL normal appearing white matter regions of interest. The boxes correspond to the interquartile ranges, wherein 50% of the *y*‐values lie. The middle horizontal line represents the median, the vertical lines represent the range of data not considered outliers, the gray dots represent the range of outliers, while the dots represent the data points (see text for statistical output).

There was no inter‐rater difference in PSR (MD = −0.15; 95% CI: −0.48 to 0.17, *p* = 0.341, Figure 2A) or R_1f_ (MD = −0.004; 95% CI: −0.01 to 0.01, *p* = 0.387, Figure 2B) values of the NAWM ROIs. The inter‐rater agreement was excellent for both PSR (Cronbach *α* = 0.98) and R_1f_ (Cronbach *α* = 0.93) values.

### 3.3 Lesion Level Analyses

A total of 2284 WM lesions (referred as T_2_‐lesions hereafter) were counted on the T_2_‐w FLAIR images of the 64 subjects who were included in the analysis. Of these, 118 were excluded because they were not visible on SWI due to susceptibility artifacts, cortical involvement, or concomitant CEL activity. Thus, 2166 T_2_‐lesions were eligible for analysis, 89 (4.1%) of which were PRLs. Fifteen of the identified PRLs were excluded due to the absence of a contralateral nPRL lesion, leaving 74 PRLs and 74 contralateral nPRLs.

**FIGURE 3:**
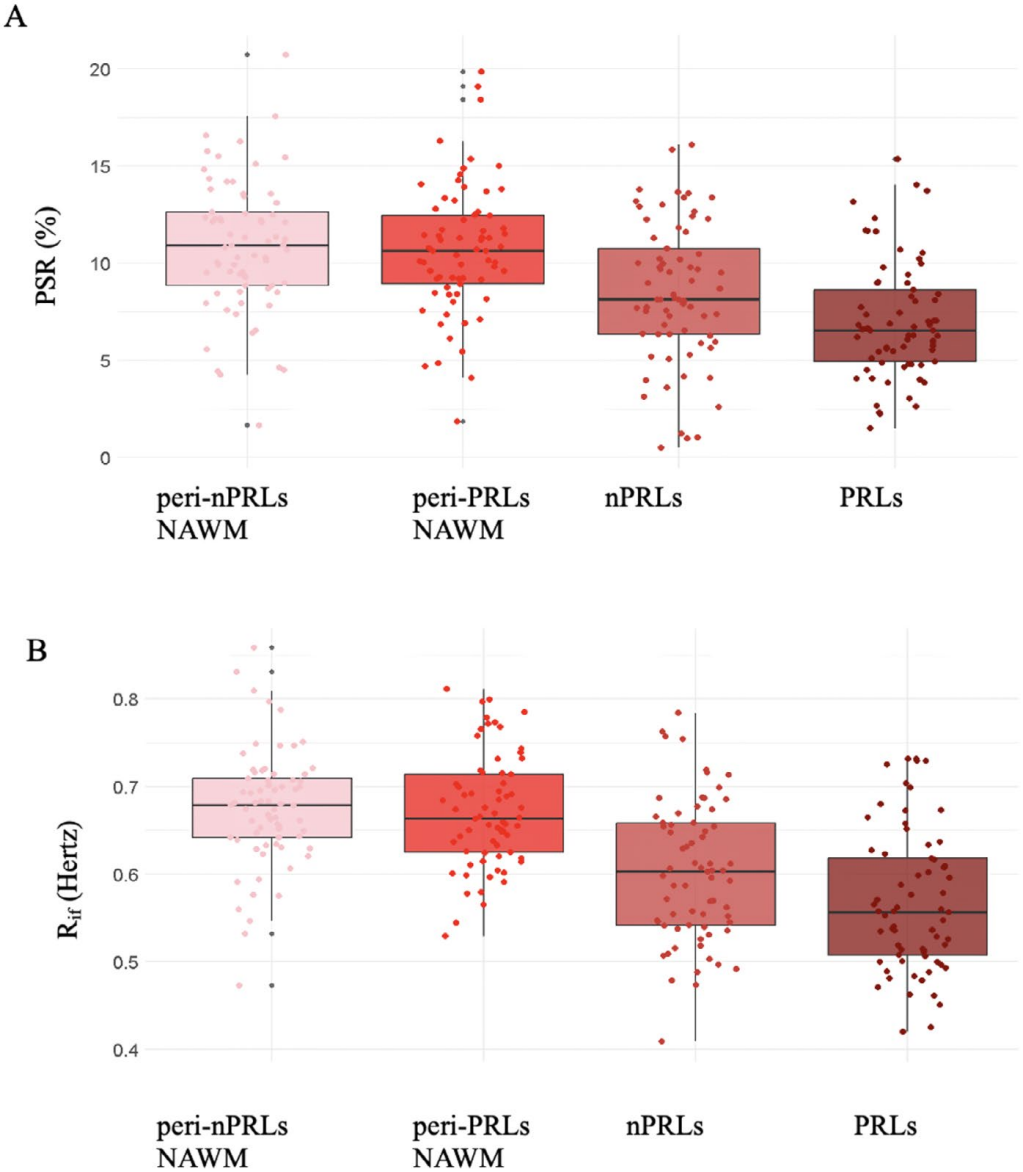
Macromolecular‐to‐free water pool‐size ratio and the spin–lattice relaxation rate of free water of different lesions and normal appearing white matter regions. Macromolecular‐to‐free water pool‐size ratio (PSR, A) and spin–lattice relaxation rate of free water (R_1f_, B) values of paramagnetic rim lesions (PRLs), non‐PRLs (nPRLs), and peri‐PRL and peri‐nPRL normal appearing white matter (NAWM) regions of interest. The boxes correspond to the interquartile ranges, wherein 50% of the *y*‐values lie. The middle horizontal line represents the median, the vertical lines represent the range of data not considered outliers, the gray dots represent the range of outliers, while the colored dots represent the datapoints (see text for statistical output).

Mean ± SD PSR (%) values were 7.15 ± 3.05 for PRLs, 8.55 ± 3.68 for nPRLs, 10.81 ± 3.59 for NAWM ROIs surrounding PRLs, and 10.90 ± 3.79 for NAWM ROIs around nPRLs. Mean ± SD R_1f_ (Hertz) values were 0.57 ± 0.08 for PRLs, 0.60 ± 0.08 for nPRLs, 0.66 ± 0.07 for NAWM ROIs surrounding PRLs, and 0.67 ± 0.07 for NAWM ROIs around nPRLs. We depict these results in Figure 3. PSR (MD = −1.40, CI = −2.27 to −0.52, *p* = 0.002) and R_1f_ (MD = −0.03, CI = −0.05 to −0.003, *p* = 0.028) values were lower in the PRLs core compared to those of the contralateral nPRLs. Both PRLs and nPRLs had lower PSR (MD = −3.66, CI = −4.23 to −3.08, *p* < 0.001 for PRLs and MD = −2.34, CI = −3.01 to −1.68, *p* < 0.001 for nPRLs) and R_1f_ (MD = −0.10, CI = −0.11 to −0.08, *p* < 0.001 for PRLs and MD = −0.07, CI = −0.09 to −0.05, *p* < 0.001 for nPRLs) values compared to the corresponding NAWM ROIs. However, there were no differences in PSR (MD = −0.09, CI = −0.85 to 0.68, *p* = 0.824) and R_1f_ (MD = 0.01, CI = −0.02 to 0.02, *p* = 0.963) values between NAWM ROIs surrounding PRLs and those around nPRLs.

**FIGURE 4:**
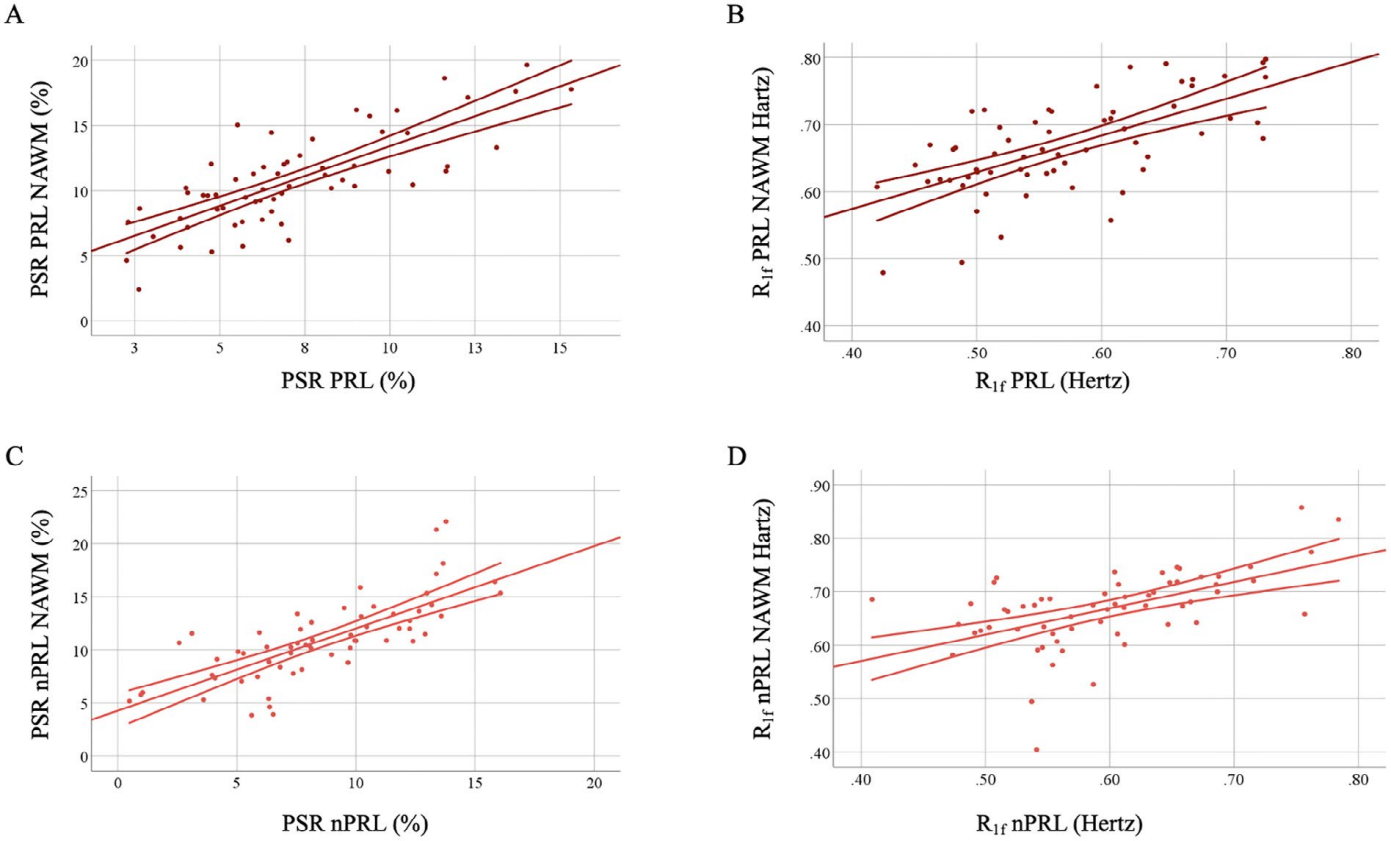
Correlations between metrics of lesion and non‐lesion tissue injury. Associative analyses showing the relationships between the following pairs: (A) macromolecular‐to‐free water pool‐size ratio (PSR) and (B) spin–lattice relaxation rate of free water (R_1f_) measured in paramagnetic rim lesions (PRLs) and corresponding normal appearing white matter (NAWM) regions of interest (ROIs), and (C) PSR and (D) R_1f_ measured in non‐PRLs and corresponding NAWM ROIs (see text for statistical output).

Positive associations were observed between the PSR (*R* ^2^ = 0.92, *p* < 0.001, Figure 4A) and R_1f_ (*R* ^2^ = 0.77, *p* < 0.001, Figure 4B) values of PRLs and those of the surrounding NAWM as well as between the PSR (*R* ^2^ = 0.77, *p* < 0.001, Figure 4C) and R_1f_ (*R* ^2^ = 0.61, *p* < 0.001, Figure 4D) values of nPRLs and those of the surrounding NAWM. A follow‐up ANOVA test demonstrated that PSR (*p* < 0.001) and R_1f_ (*p* < 0.001) of PRLs explained a larger degree of variance of the same metrics measured in the surrounding NAWM than that explained by nPRL PSR/R_1f_ values for the surrounding NAWM.

### 3.4 Subject Level Analyses

**FIGURE 5:**
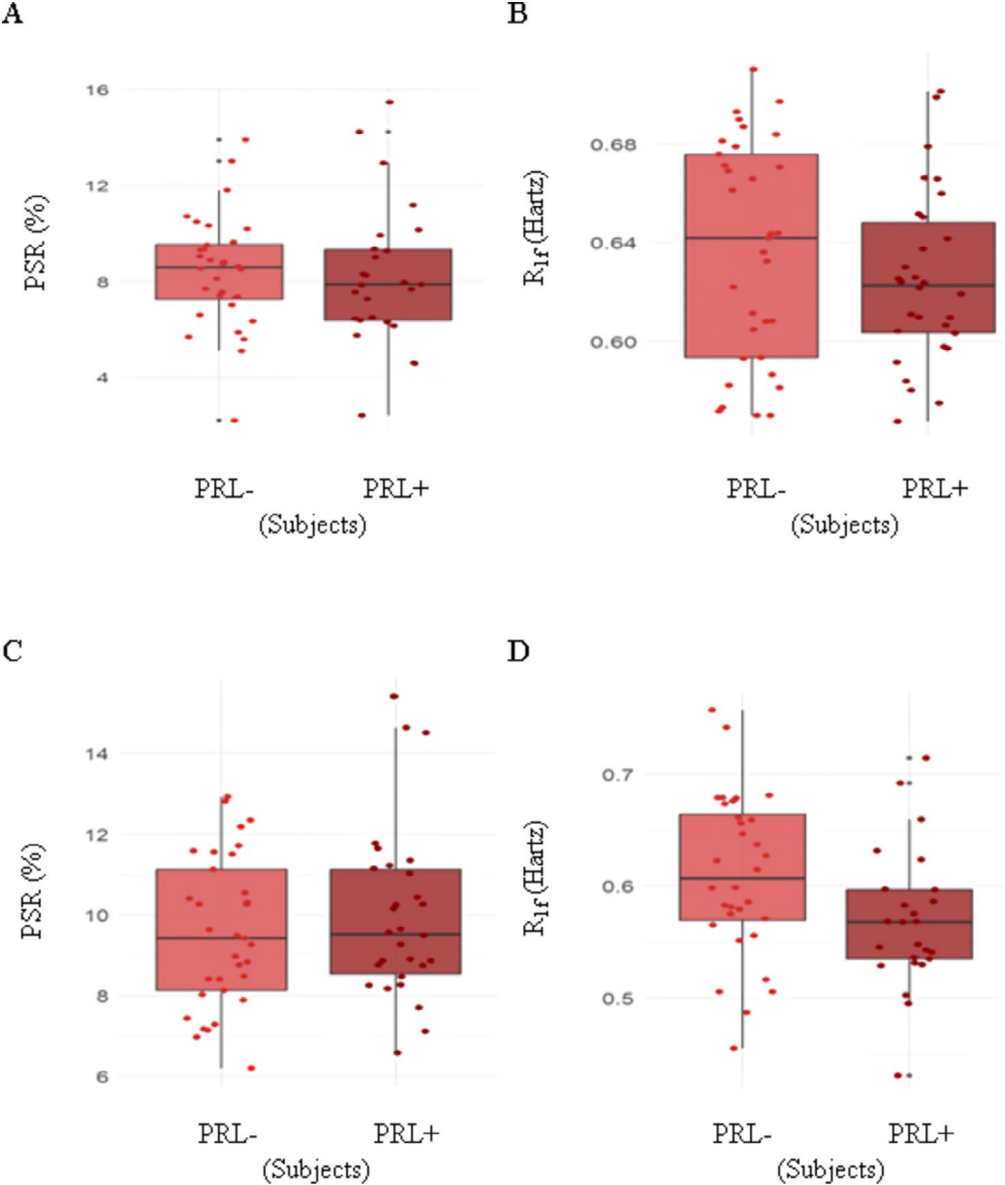
Differences between people with and without paramagnetic rim lesions. Macromolecular‐to‐free water pool‐size ratio (PSR, A and C) and spin–lattice relaxation rate of free water (R_1f_, B and D) values measured in the whole brain normal appearing white matter (A and B) and in non‐paramagnetic rim lesions (nPRL) (C and D) of people with (PRL+) and without (PRL−) PRLs. The boxes correspond to the interquartile ranges, wherein 50% of the *y*‐values lie. The middle horizontal line represents the median, the vertical lines represent the range of data not considered outliers, the gray dots represent the range of outliers, while the colored dots represent the data points (see text for statistical output).

PRLs were identified in 29 (42.6%) of the 68 subjects, although as stated earlier the analyses herein presented were perfroemd in 64 participants. PRL+ people had similar PSR (*p* = 0.434, Figure 5A) and R_1f_ (*p* = 0.267, Figure 5B) values of the whole brain NAWM. There were no group differences in T_2_‐lesions PSR values (*p* = 0.764, MD = −0.20, CI = −1.60 to 1.18, Figure 5C). Lower T_2_‐lesions R_1f_ (*p* = 0.030, MD = −0.04, CI = −0.08 to −0.01, Figure 5D) values were instead seen in PRL+ patients.

## 4 Discussion

Our results confirm that, as seen later in the disease course [35, 36, 37], PRLs exhibit signs of more aggressive tissue injury at the time of disease diagnosis as well, as indicated by lower PSR and R_1f_ values compared to nPRLs. However, contrary to previous findings [35, 36, 37], no difference in the degree of tissue injury was observed between peri‐PRL and peri‐nPRLs NAWM. Furthermore, PRL+ people showed similar degrees of pathology of the whole NAWM but some differences in the degree of WM lesions structural damage when compared to PRL− ones.

With respect to PRL and nPRL core metrics, our results align with existing literature in people with more advanced MS assessed using various advanced imaging techniques to assess PRLs, at both 3 T and 7 T [25, 35, 36, 38, 39, 40]. In these cohorts, relative to nPRL cores, PRL cores were found to have lower magnetization transfer ratio [38], longer T_1_ relaxation times [35, 36, 39], slower R2* relaxation rate [39], and lower myelin water fraction [40], PSR [25], and neurite density index [40]. We here confirm that PRLs have longer T_1_ relaxation times (1/R_1f_) and lower PSR values. As an expansion of previously reported results in a subgroup of our study cohort [25], current findings assert the novel concept that differences in tissue integrity between PRLs and nPRLs are present as early as the time of disease diagnosis and do not necessarily mature as the disease progresses. Early in the disease course, PRL injury is characterized by a higher degree of myelin loss as measured using PSR but also some degree of water replacement of tissue, potentially secondary to axonal loss, as measured by the R_1f_ (1/T_1_). This may signify that the more aggressive disease pathology seen in PRLs could be present very early, perhaps even at the time of lesion formation.

Our results on the peri‐lesional NAWM are instead not in alignment with previous in vivo [36, 37] and post‐mortem [35] studies conducted in cohorts of people with more advanced MS. Krajnc and collaborators [36] measured longer T_1_ relaxation times in NAWM surrounding PRLs relative to that surrounding nPRLs, more so in people with secondary progressive (SP) MS than relapsing remitting (RR) MS. Mohebbi and collaborators [37] used tractography to demonstrate that tracts connected to PRLs had significantly lower fractional anisotropy (FA) compared to those connected to non‐PRLs, indicating that PRLs are more destructive to their surrounding tissue compared to nPRLs. These in vivo findings were corroborated by previous combined MRI‐histology data proposed by DalBianco and collaborators [35]. In this study, a T_2_‐hyperintense signal was seen around PRLs and demonstrated to correspond to areas of reduced axonal density and increased axonal spheroids, relative to NAWM surrounding nPRL or remyelinated plaques [35].

The discrepancy with these three studies can likely be attributed to differences in cohort disease stages as well as methodological divergences. Krajnc and collaborators [36] studied a mixed cohort of 30 people with RRMS and SPMS and a median disease duration of 12 years and used multi dynamic multi echo imaging to identify peri‐lesional ROIs on the T_1_ map. Similarly, Mohebbi and collaborators [37] focused on a cohort of 115 people with RRMS and PMS and a median disease duration of 17 years. This study relied on tractography to compare PRL‐connected WM tracts to those not connected to PRLs, focusing on global tract integrity metrics such as FA, mean diffusivity (MD), and RD. Importantly, their analysis was not confined to peri‐lesional ROIs but also captured global alterations in the WM associated with lesion connectivity. Finally, the autopsy cases analyzed by DalBianco and collaborators [35] had a mean disease duration of 31.5 years. This contrasts with our study cohort, formed by nearly diagnosed MS and even CIS or RIS at the time of the MRI. The longer the diseases duration, the older the age of lesions, a factor that is important in the context of peri‐plaque disease formation. Furthermore, our study employed a rigorous anatomical matching approach within each subject, identifying PRLs that were spatially and morphologically matched to nPRLs and drawing adjacent NAWM ROIs equidistantly and cautiously to avoid partial volume effects. Thus, our anatomically controlled methodology in an early MS cohort may explain why we did not observe the same degree of peri‐lesional tissue injury as reported in cohorts with more advanced disease and different methodological frameworks.

Peri‐lesional disease injury is thought to be secondary to Wallerian degeneration spreading out from the lesion core and inducing secondary demyelination [41]. The degree of Wallerian degeneration is related to that of tissue injury with the lesion core [3, 10]. Accordingly, we did find that lesional PSR and R_1f_ values measured in the lesion cores were associated with those measured in the surrounding NAWM, more so for PRLs than for nPRLs. Nonetheless, Wallerian degeneration is a slow process [41] that evolves over the course of years, and it is unlikely to be measurable early in the disease course. This conclusion is also in line with the finding of Krajnc and collaborators [36] showing that T_1_ relaxation times measured around PRLs were significantly longer in people with SPMS relative to those with RRMS [36]. Notably, the former people were older and had a longer disease duration than the latter ones. Our group is monitoring this cohort longitudinally, and this assessment will further characterize the dynamics of perilesional tissue damage.

We did not find any differences in the degree of whole brain NAWM structural injury between PRL+ and PRL− people. No data in the literature are available to challenge our findings, and future studies are warranted to corroborate or disprove them. Both PSR and R_1f_ values were entirely overlapping between the two groups, likely because of an overall low pathological heterogeneity at this stage of the disease. It is plausible that the degree of NAWM injury is at this stage small [42] and limited to a few cases only, making differences between PRL+ and PRL− individuals difficult to detect. However, it is also possible that PRLs are not associated with a widespread NAWM pathology measurable with our employed methods, indicative of myelin loss and neurodegeneration and unable to capture metrics of NAWM chronic inflammation.

When looking at differences in PSR and R_1f_ of all WM lesions between PRL+ and PRL− subjects, we observed that WM lesions of the former subjects had lower R_1f_ but similar PSR values. R_1f_ is quite a non‐specific measure as predominantly indicative of water content changes. In lack of concomitant PSR differences, we postulate that changes in R_1f_ might be due to microedema potentially secondary to chronic inflammation. To this end, we note that our T_2_‐lesion counts excluded PRLs but also CELs, to include only non‐active lesions as definable by conventional MRI. In this regard, our results would be in line with those recently reported by Treaba and collaborators [43] who found a correlation between the count of PRLs and that of nPRLs but chronically active as measured by ^11^C‐PBR28 positron emission tomography. This is an intriguing finding which could underscore the fact that PRL are likely sentinel of more widespread chronic inflammatory disease, potentially explaining the smoldering progression and symptoms of the disease [44].

### 4.1 Study Limitations and Conclusions

The small sample size for such a homogeneous cohort of pwMS, pwCIS, and pwRIS early in the disease course is an important limitation of our study. As stated, this factor likely masked several differences and prevented subgroup analysis for pwCIS and pwRIS. Consequently, data from all disease phenotypes were processed together. However, it is possible that different disease phenotypes exhibit distinct relationships in PSR and R_1f_ values, and assessing it could provide deeper insights into the underlying mechanisms by which PRLs affect disease outcome over time.

Despite these limitations, we propose the largest 7 T study performed in pwMS thus far. We show that early in MS, the presence of PRLs is not associated with measurable differences in the degree of tissue damage between NAWM surrounding PRLs and nPRLs. This association likely becomes more measurable as the disease advances, in line with the dynamic and relentless nature of MS and with the correlations seen between the degree of lesional and surrounding NAWM injury. We also show that WM lesions of PRL+ people with MS, CIS, or RIS show features of more advanced tissue injury. The pathobiology of this injury remains to be better characterized but adds to the knowledge that PRL+ individuals have an overall more aggressive disease presentation.

## Author Contributions

E.M., A.A.T., and F.B. conceptualized the study and designed the framework. T.V., C.G., C.K., Z.R., J.K., R.K., and F.B. contributed to clinical data collection, M.R.I. acquisition, and patient cohort organization. J.W., C.J., J.X., and I.O. assisted with implementing image processing pipelines. E.M., A.A.T., J.W., H.F.K., B.H., M.A.C., R.C., R.K., and V.K. were involved in data collection. E.M., A.A.T., J.W., and F.B. supported with data analysis and statistical interpretation. F.B. provided project oversight, accountability, and final approval. E.M., A.A.T., and F.B. drafted the initial manuscript. All authors reviewed, revised, and approved the final manuscript.

## Conflicts of Interest

The authors declare no conflicts of interest.

## Data Availability

Data are available to share upon reasonable request to the corresponding author.
